# Characteristics and cardiovascular complications of a large cohort of adults diagnosed with type 2 diabetes <45 years

**DOI:** 10.1186/s13098-017-0227-z

**Published:** 2017-05-02

**Authors:** Barbara Deconinck, Chantal Mathieu, Katrien Benhalima

**Affiliations:** Department of Endocrinology, UZ Gasthuisberg, KU Leuven, Herestraat 49, 3000 Leuven, Belgium

**Keywords:** Young-onset type 2 diabetes, Cardiovascular risk factors, Complications, Management

## Abstract

**Background:**

The aim was to evaluate the characteristics and cardiovascular complications of a large Belgian cohort of adults diagnosed with type 2 diabetes (T2DM) <45 years.

**Methods:**

Retrospective analysis of 886 patients diagnosed with T2DM <45 years and 933 T2DM patients diagnosed at the age between 60 and 70 years. To compare variables between groups, the independent *t* test or paired *t* test was used for normally distributed continuous variables, the Mann–Whitney’s *U*-test for non-normally distributed continuous variables and the Chi squared test or McNemar test for categorical variables. Multivariable logistic regression was used to adjust for confounders.

**Results:**

In the young-onset T2DM cohort, the age at diagnosis was 37.3 ± 6.4 years, 44.1% of patients were female and 12.1% were from an ethnic minority (EM) background. At last visit, age of patients was 57.3 ± 12.5 years with a diabetes duration of 20.5 ± 11.8 years and a mean HbA1c of 7.3% ± 1.3 (56 mmol/mol ± 14). Of all patients, 56.8% were obese, 49.9% were hypertensive, 34.1% did not reach the LDL cholesterol target and 20.1% had a cardiac event by time of last visit. Compared to women, men had a higher HbA1c [7.3% ± 1.4 (56 mmol/mol ± 15) vs. 7.1% ± 1.2 (54 mmol/mol ± 13), p = 0.021] and a significantly higher rate of cardiac events, even after adjustment for confounders (24.3 vs. 14.8%, p = 0.010). Compared to Caucasians, EM patients were younger at diagnosis (35.4 ± 6.8 years vs. 37.6 ± 6.2 years, p = 0.001) and were less often obese (43.3 vs. 55.6%, p = 0.007). Compared to the first visit, glycemic control improved [7.3% ± 1.3 (56 mmol/mol ± 14) vs. 7.9% ± 1.7 (62 mmol/mol ± 19), p < 0.0001] by the time of the last visit. Compared to the older-onset T2DM cohort, young-onset T2DM patients showed a higher HbA1c [7.3 ± 1.3% (56 mmol/mol ± 14) vs. 6.9 ± 1.0% (51 mmol/mol ± 11), p = <0.0001] and a higher BMI (31.2 ± 5.8 vs. 29.6 ± 5.5 kg/m^2^, p = <0.0001) at last contact. When adjusted for age, diabetes duration, HbA1c and cardiovascular risk factors, there was no difference in cardiovascular events between the two cohorts.

**Conclusions:**

A diagnosis of T2DM <45 years has an important impact on patients’ lives. Prevention measures are essential, but also specific attention to this high-risk group is needed for them to better achieve their therapeutic targets.

## Background

The incidence of young-onset type 2 diabetes (T2DM) is increasing worldwide and is strongly associated with the rise of obesity [1]. In the past, T2DM was considered a disease of older adults, but we are now also seeing the condition in children, adolescents and young adults [2, 3]. Most evidence for an epidemic of young-onset T2DM comes from studies in the USA and Asia (particularly Japan) [4]. The ‘SEARCH for Diabetes in Youth study’ in the USA has reported incidence and prevalence rates of 3.7–19/100.000 per year and 0.18–1.06/1.000 respectively, with higher rates seen in black and other ethnic minority groups [5, 6]. More importantly, studies show that the prevalence is increasing over time [7]. Data from European populations are more limited and most data have been retrieved from pediatric care units (<17 years). Data from large diabetes centers in Sheffield and Leicester in the UK have shown that about 20% of adults with diabetes diagnosed before the age of 40 years have T2DM [8, 9]. The definition of young-onset T2DM remains variable. Generally, subjects are classified as young-onset T2DM if they were diagnosed with T2DM <45 years. This cut-off point has been selected for three reasons. Firstly, 45 years is the age the American Diabetes Association currently recommends for T2DM screening in adults. Secondly, the hazard of developing a myocardial infarction is fourfold higher in T2DM diagnosed <45 years compared to T2DM diagnosed >45 years [10]. Thirdly, using a cut-off point <45 years will include women of childbearing age, a cohort that requires special consideration [3].

Despite the younger age, recent data report that young-onset T2DM patients have a more severe phenotype, a more adverse cardiovascular risk profile and a poorer glycemic control even with a higher use of insulin therapy [10]. As a result, young-onset T2DM patients are at a high lifetime risk for the development of micro-and macrovascular complications [7]. However, few studies have evaluated the long-term risk for cardiovascular complications in European young-onset T2DM patients. We therefore performed a retrospective study of patients diagnosed with T2DM <45 years attending our university hospital between 2004 and 2014. We hypothesized that patients with young-onset T2DM have a high rate of cardiovascular complications despite a rather young age. We evaluated the general characteristics, cardiovascular risk factors, micro- and macrovascular complications and management strategies over time in a large Belgian young-onset T2DM cohort. Moreover, we compared the general characteristics of this population with a cohort of T2DM patients diagnosed between 60 and 70 years.

## Methods

We performed a retrospective analysis of the electronic medical files of all patients who attended our center at the University Hospital UZ Leuven in Belgium between 01-01-2004 and 31-12-2014 and who had the diagnosis of T2DM <45 years. The study was approved by the Institutional Review Board of UZ Leuven (S58334) and conducted according to the principles expressed in the Declaration of Helsinki. Due to the retrospective nature of the study there was no need for informed consent from the participants (the data were analyzed anonymously) as in compliance with the Belgian Law of 8 December, 1992 on the protection of privacy and the Belgian Law of 22 August, 2002 on the rights of the patient.

Leuven is a medium-size city in the Dutch-speaking part of Belgium and has a population with a slightly lower ratio of ethnic minorities (EM) (15.4% in 2011) compared to the larger Belgian cities [11]. Of the overall Belgian adult population, 48% is overweight and 13% is obese (2013) [12]. The background prevalence of T2DM in Belgium is 6.5% [13]. UZ Leuven is one of the largest hospitals in the country. The out-patient diabetes center attends to about 4400 T2DM patients and 1400 type 1 diabetes (T1DM) patients yearly. Patients are under the care of an experienced multidisciplinary team of endocrinologists, diabetes nurses, dieticians and psychologists. A well-structured electronic medical file, specifically designed for the diabetes center, is used for routine care.

Subjects who attended the diabetes center between 2004 and 2014 were identified by the electronic medical database. A total of 9072 patients with diabetes were identified of whom 5968 had T2DM (65.8%), 1968 had T1DM (21.7%), 648 had secondary diabetes (7.1%), 140 had gestational diabetes (1.5%), 239 had another type of diabetes (2.6%) and 69 patients had a metabolic syndrome (0.8%). To ensure that only patients with established T2DM were included, we excluded patients with only one contact labeled as T2DM between 2004 and 2014. Of the remaining 4557 patients with T2DM, 1077 patients were identified being younger than 45 years at the time of diagnosis. T2DM accounted for 13.1% of the diabetes population <45 years attending our diabetes center in 2014. Due to suspicion of misdiagnosis, 86 files were evaluated in more detail and 54 patients were further excluded due to misclassification. Since the aim was also to assess the evolution over time in this young-onset cohort, we retrieved details of the first and last visit in our center, ranging from 1998 to 2015. Another 137 patients were excluded because the follow-up time between the two visits was less than one year, leaving a young-onset T2DM cohort of 886 patients. To be able to compare the general characteristics of the young-onset T2DM adults with T2DM patients diagnosed at an older age, we also retrospectively evaluated the records of 933 patients who attended UZ Leuven between 2004 and 2014 and who had been diagnosed with T2DM at an age between 60 and 70 years.

Outcomes were obtained from review of the electronic database. Clinical data recorded were gender, age, nationality, ethnicity, past history, length, weight, BMI (kg/m^2^), blood pressure (BP), diabetes duration, presence of diabetic retinopathy, smoking status, medication use and use of insulin. Biochemical data recorded were HbA1c, total cholesterol, LDL-cholesterol, triglycerides and microalbuminuria (defined as a urine albumin-creatinin ratio >30 mg/g creatinin or a 24 h excretion of urinary albumin of 31–299 mg or a urinary concentration of microalbumin of 21–199 mg/l). Cardiovascular risk factors recorded were hypertension (systolic BP ≥ 140 and/or diastolic BP ≥ 90 mmHg), overweight (BMI 25–29.9 kg/m^2^) or obesity (BMI ≥ 30 kg/m^2^), nicotine use, LDL ≥ 70 mg/dl in secondary cardiovascular prevention, LDL ≥ 100 mg/dl in primary cardiovascular prevention and a triglyceride level ≥150 mg/dl. The cardiovascular complications recorded were carotid surgery, surgery of the lower limbs [thrombolysis, balloon dilatation, percutaneous transluminal arterioplasty (PTA) or an open vessel operation], transient ischemic attack (TIA) or cerebrovascular accident (CVA) and cardiac events [myocardial infarction or an intervention such as a percutaneous transluminal coronary angioplasty (PTCA) or a coronary artery bypass graft (CABG)]. When the file mentioned a diagnosis of ‘cardiac angina’ or ‘limb claudication’, it was analyzed in more detail to establish whether the patient had undergone an intervention or had a history of CVA or myocardial infarction. Furthermore, we analyzed the number of re-interventions, defined as new interventions following a previously conservative approach or a second TIA/CVA/cardiac event between the first and the last contact.

During the study time period, HbA1c was measured using different techniques. Between May 2002 and August 2007, the immunoassay method Dimension HA1C of Dade Behring was used. From August 2007 until October 2015, the reversed-phase cation-exchange chromatography was used (ADAMS HA-8160, Menarini Diagnostics Benelux). Since October 2015, HbA1c has been measured by a high performance liquid chromatography principle with the cationic non-porous ion exchanger using the ionic difference (Tosoh HLC-723G8, Tessenderlo, Belgium). Since 1996, reproduction of Hba1c complies with the National Glycohemoglobin Standardization Program [14]. During the entire period of the study, lipids and microalbuminuria were measured by the same biomedical technique with only a few changes in the calibration methods/equipment/assays. Total cholesterol was measured on Cobas 8000, Roche Diagnostics by enzymatic colorimetic CHOD-PAP method, triglycerides by enzymatic colorimetic GPO-PAP method, HDL cholesterol by dextran sulfate/Mg precipitation followed by enzymatic colorimetric CHOD-PAP reaction, calculated LDL-cholesterol by Friedewald formula (Total cholesterol—HDL cholesterol—Triglycerides/4). Microalbuminuria was measured by an immuno-turbidimetric method (Tina-Quant Albumin) on a Cobas 8000 auto-analyzer from Roche Diagnostics.

### Statistical analyses

Statistical analyses were performed using SPSS 23. Continuous variables were presented as mean and standard deviation if normally distributed and as median otherwise. Categorical variables were expressed as percentage. To compare variables between two groups, the independent *t* test or paired *t* test was used for normally distributed continuous variables, the Mann–Whitney’s *U*-test for non-normally distributed continuous variables and the Chi squared test or McNemar test for categorical variables. For comparison of the occurrence of cardiovascular events between groups, logistic regression analysis was performed to adjust for confounding factors such as smoking, HbA1c, BMI, LDL-cholesterol, systolic and diastolic blood pressure, diabetes duration, age and microalbuminuria. A p value of <0.05 (two-tailed) is considered significant.

## Results

### Characteristics of the young-onset T2DM cohort at last contact

#### General characteristics

Of the 886 patients, mean age at diagnosis was 37.3 ± 6.4 years, 44.1% were women and 12.1% (106) were from an EM background (32.0% Northern-African, 29.2% black African, 19.8% Asian, 16.0% Middle-Eastern and 2.8% South-American). Mean age at last contact was 57.3 ± 12.5 years and mean diabetes duration was 20.5 ± 11.8 years. Mean HbA1c was 7.3% ± 1.3 (56 mmol/mol ± 14) with 45.5% of patients reaching an HbA1c <7.0% (52 mmol/mol) and 81.9% receiving insulin. Mean BMI was 31.2 ± 5.8 kg/m^2^, with 32.4% of patients being overweight and 56.8% being obese. Furthermore, 16.6% of patients smoked, 49.9% were hypertensive, 33.3% had elevated triglycerides and 34.1% did not reach the LDL cholesterol target. In addition, 81.7% of patients had at least two or more cardiovascular risk factors. By the time of the last contact, 0.9% of patients (8) had received carotid surgery, 6.9% of patients (61) had received vascular surgery of the lower limbs, 7.2% of patients (64) had experienced a CVA and 20.1% of patients (178) had suffered from a cardiac event. Diabetic retinopathy was present in 40.2% and microalbuminuria in 34.4% of patients.

#### Comparison between women and men

Table 1 gives an overview of the differences between women and men at the time of the last contact. Men were older at diagnosis (38.1 ± 5.8 vs. 36.4 ± 6.9, p < 0.0001), had a higher HbA1c [7.3% ± 1.4 (56 mmol/mol ± 15) vs. 7.1% ± 1.2 (54 mmol/mol ± 13), p = 0.021], had lower rates of obesity (51.2 vs. 64.1%, p < 0.0001) but higher rates of hypertension (53.4 vs. 45.3%, p = 0.019) and smoked more often (20.2 vs. 12.0%, p = 0.001).

**Table 1:** Characteristics and complications for men and women at last contact in the young-onset cohort

	Men N = 495 (55.9%)	Women N = 391 (44.1%)	p value
% EM	12.4	11.7	0.762
Mean age at diagnosis	38.1 ± 5.8	36.4 ± 6.9	*<0.0001*
Mean age at last visit (years)	56.9 ± 11.5	57.8 ± 13.8	0.283
Mean follow-up (years)	8.1 ± 4.8	8.8 ± 5.1	*0.029*
Mean diabetes duration (years)	19.3 ± 11.0	22.0 ± 12.7	*0.001*
Mean HbA1c % (mmol/mol)	7.3 ± 1.4 (56 ± 15)	7.1 ± 1.2 (54 ± 13)	*0.021*
% HbA1c ≥9% (74 mmol/mol)	13.7	8.0	*0.008*
% Statin	79.6	70.8	*0.006*
Number of antihypertensive medications			0.177
0	21.4	25.3	
1	18.4	15.3	
2	17.6	23.0	
≥3	42.5	36.4	
% Aspirin	58.6	55.5	0.356
% MF	53.1	55.5	0.483
% SU	1.4	1.8	0.656
% MF + SU	16.6	8.2	*<0.0001*
% Glitazones	0.2	0.5	0.431
% DPP-4	3.6	3.3	0.802
% GLP-1	10.9	5.1	*0.002*
% Insulin	79.8	84.7	0.062
% On 1 injection of insulin	5.3	5.6	0.807
% On 2 or 3 injections of insulin	5.9	8.2	0.175
% On multiple injections of insulin	68.7	70.8	0.488
Mean BMI (kg/m^2^)	30.5 ± 4.9	32.1 ± 6.7	*<0.0001*
% Overweight	39.4	23.4	*<0.0001*
% Obese	51.2	64.1	*<0.0001*
% Hypertension	53.4	45.3	*0.019*
Mean TG	162.9 ± 175.7	135.6 ± 78.8	*0.007*
Mean LDL	74.1 ± 30.1	81.7 ± 31.2	*<0.0001*
% High TG	35.7	30.1	0.093
% LDL not on target in primary CV prevention	24.8	32.4	0.065
% LDL not on target in secondary CV prevention	51.1	39.9	0.052
% Smoking	20.2	12.0	*0.001*
% ≥3 CV risk factors	59.4	50.1	*0.006*
% DRP	40.3	40.1	0.942
% Microalbuminuria	40.6	26.2	*0.001*
Mean age first CV event	53.4 ± 9.5	57.9 ± 10.1	*<0.0001*
% New surgery lower limbs	3.0 (15)	2.0 (8)	
% New TIA/CVA	1.0 (5)	1.0 (4)	
% New cardiac event	4.8 (24)	3.1 (12)	

Data are mean ± SD, % or (number)

p values highlighted in italic are considered significant

*EM* ethnic minority; *MF* metformin with or without insulin; *SU* sulfonylureum with or without insulin; *DPP-4* DPP-4-inhibitor; *GLP-1* GLP-1-receptor-agonist; *BMI* body mass index; *TG* triglycerides; *LDL* LDL-cholesterol; *CV* cardiovascular; *DRP* diabetic retinopathy; *TIA* transient ischemic attack; *CVA* cerebrovascular accident; *cardiac event* a myocardial infarction or an intervention such as a percutaneous transluminal coronary angioplasty or a coronary artery bypass graft

#### Comparison between Caucasians and EM patients

Compared to Caucasians, EM patients were younger at diagnosis (35.4 ± 6.8 vs. 37.6 ± 6.2 years, p = 0.001), had a shorter diabetes duration at last visit (15.8 ± 10.6 vs. 21.2 ± 11.9 years, p = 0.002) and were less often obese (43.3 vs. 55.6%, p = 0.007) (Table 2).

**Table 2:** Characteristics and complications for Caucasians and EM patients at last contact in the young-onset cohort

	Caucasian N = 769 (87.9%)	EM N = 106 (12.1%)	p value
% Men	56.0	57.5	0.762
Mean age at diagnosis	37.6 ± 6.2	35.4 ± 6.8	*0.001*
Mean age at last visit (years)	58.4 ± 12.4	50.8 ± 11.7	*<0.0001*
Mean follow-up (years)	8.6 ± 4.9	7.0 ± 4.8	*0.002*
Mean diabetes duration (years)	21.2 ± 11.9	15.8 ± 10.6	*0.002*
Mean HbA1c % (mmol/mol)	7.3 ± 1.3 (56 ± 14)	7.4 ± 1.4 (57 ± 15)	0.461
% Statin	78.1	59.4	*<0.0001*
Number of antihypertensive medications			
0	20.2	42.5	*<0.0001*
1	16.3	23.6	
2	21.0	11.3	
≥3	42.5	22.7	
% MF	53.1	62.3	0.077
% SU	1.4 (11)	2.8 (3)	0.283
% MF + SU	12.9	11.3	0.649
% Glitazones	0.4	0	0.519
% DPP-4	2.9 (67)	7.5 (4)	*0.013*
% GLP-1	8.7	3.8	0.080
% Insulin	81.4	86.8	0.174
% On 1 injection of insulin	5.3	4.7	0.788
% On 2 or 3 injections of insulin	7.2	5.7	0.570
% On multiple injections of insulin	68.9	76.4	0.113
Mean BMI (kg/m^2^)	31.4 ± 5.9	29.8 ± 4.6	*0.009*
% Overweight	28.8	44.3	*0.002*
% Obese	55.6	43.3	*0.007*
% Hypertension	48.7	43.4	0.269
Mean TG	151.7 ± 144.7	144.8 ± 132.7	0.660
Mean LDL	76.7 ± 31.0	82.8 ± 29.7	0.075
% High TG	30.7	29.2	0.913
% LDL not on target in primary CV prevention	27.2	30.9	0.472
% LDL not on target in secondary CV prevention	45.2		
% Smoking	17.2	12.3	0.201
% ≥3 CV risk factors	56.6	45.3	*0.027*
% DRP	40.8	40.4	0.950
% Microalbuminuria	35.1	32.1	0.667
% New surgery lower limbs	3.0 (23)	0 (0)	
% New TIA/CVA	1.0 (8)	0.9 (1)	
% New cardiac event	4.3 (33)	2.8 (3)	

Data are mean ± SD, % or (number)

p values highlighted in italic are considered significant

*EM* ethnic minority, *MF* metformin with or without insulin; *SU* sulfonylureum with or without insulin; *DPP-4* DPP-4-inhibitor; *GLP-1* GLP-1-receptor-agonist; *BMI* body mass index; *TG* triglycerides; *LDL* LDL-cholesterol; *CV* cardiovascular; *DRP* diabetic retinopathy; *TIA* transient ischemic attack; *CVA* cerebrovascular accident; *cardiac event* a myocardial infarction or an intervention such as a percutaneous transluminal coronary angioplasty or a coronary artery bypass graft

#### Comparison between age of diagnosis <35 years and age at diagnosis between 35 and 45 years

Data were generally comparable between the two age groups, except for a lower proportion of men (48.6 vs. 58.7%, p = 0.006), less frequent use of statins (65.5 vs. 79.9%, p < 0.0001) and anti-hypertensive medication (p < 0.0001), more frequent use of insulin (88 vs. 79.6%, p = 0.004) and a higher LDL-cholesterol (84.6 ± 34 vs. 74.7 ± 29, p < 0.0001) in the group with age of diagnosis <35 years compared to the group diagnosed at an age between 35 and 45 years.

#### Cardiovascular events at last visit in the different subgroups in the young-onset T2DM cohort

In the unadjusted analyses, there were statistically significant more cardiac events in men, Caucasians and the group with age at diagnosis between 35 and 45 years. However, after adjustment for confounders such as smoking, HbA1c, BMI, LDL-cholesterol, systolic and diastolic BP, diabetes duration, age and microalbuminuria, rates of cardiac events remained only significantly higher in men compared to women (24.3 vs. 14.8%, p = 0.010) (Table 3).

**Table 3:** Overview of the cardiovascular events at last contact in the different young-onset T2DM subgroups

	Carotid surgery	Surgery lower limbs	TIA/CVA	Cardiac event	CV event
Men vs. women					
% Men	0.8 (4)	8.3 (41)	8.5 (42)	24.3 (120)	41.4 (205)
% Women	1.0 (4)	5.1 (20)	5.6 (22)	14.8 (58)	33.2 (130)
p value	0.737	0.064	0.103	*<0.0001*	*0.013*
Adjusted p value	0.938	0.154	0.149	*0.010*	*0.014*
Caucasian vs. EM					
% Caucasians	1.0 (8)	7.7 (59)	7.9 (61)	21.6 (166)	40.8 (313)
% EM	0 (0)	1.9 (2)	2.8 (3)	10.4 (11)	17.9 (19)
p value	0.291	*0.028*	0.058	*0.0008*	*<0.0001*
Adjusted p value	1.000	0.845	0.998	0.960	0.926
Different age groups at diagnosis					
% Diagnosis <35 years	0.8 (2)	6.4 (16)	3.6 (9)	15.7 (39)	26.9 (67)
% Diagnosis 35–45 years	0.9 (6)	7.1 (45)	8.6 (55)	21.9 (139)	42.1 (268)
p value	0.844	0.736	*0.009*	*0.038*	*<0.0001*
Adjusted p value	0.996	0.566	0.744	0.651	0.189

Data are % or (number)

p values highlighted in italic are considered significant

*TIA* transient ischemic attack; *CVA* cerebrovascular accident; *CV* cardiovascular; *EM* ethnic minority; *cardiac event* a myocardial infarction or an intervention such as a percutaneous transluminal coronary angioplasty or a coronary artery bypass graft

### Comparison between the first and last visit of the young-onset T2DM cohort

Compared to the first visit, by the time of the last visit, glycemic control had improved [7.3% ± 1.3 (56 mmol/mol ± 14) vs. 7.9% ± 1.7 (62 mmol/mol ± 19), p < 0.0001] in line with more use of oral antidiabetic drugs (OAD) and insulin (81.9 vs. 36.9%, p < 0.0001) (Table 4). Furthermore, BMI decreased (31.2 ± 5.8 vs. 31.7 ± 5.8, p = 0.009), there was a lower LDL-cholesterol (77.9 ± 31.1 vs. 107.3 ± 37.4, p < 0.0001) and less hypertension (49.9 vs. 55.6%, p = 0.018). At last contact, the rate of cardiovascular events increased (37.8 vs. 21.6%, p < 0.0001). Over a mean follow-up of 8.4 ± 4.9 years, 2.6% (23) of patients underwent a second surgery of the lower limbs, 1.0% (9) had a new CVA and 4.1% (36) had a new myocardial infarction or cardiac intervention (Table 4).

**Table 4:** Characteristics and complications for the first and last visit of the young-onset T2DM cohort

		First contact N = 886	Last contact N = 886	p value
Mean age at contacts		48.9 ± 11.5	57.3 ± 12.5	*<0.0001*
Mean diabetes duration (years)		12.1 ± 10.7	20.5 ± 11.8	*<0.0001*
Mean HbA1c % (mmol/mol)		7.9 ± 1.7 (62 ± 19)	7.3 ± 1.3 (56 ± 14)	*<0.0001*
% HbA1c ≥7% (52 mmol/mol)		66	54.5	*<0.0001*
% Statin		32.2	75.7	*<0.0001*
Number of antihypertensive medications				*<0.0001*
0		44.9%	23.1%	
1		21.7%	17.0%	
2		17.7%	20.0%	
≥3		15.8%	39.7%	
% MF		30.9	54.2	*<0.0001*
% SU		5.5	1.6	*<0.0001*
% MF + SU		20.0	12.9	*<0.0001*
% DPP-4		1.7	3.5	*0.014*
% GLP-1		1.2	8.4	*<0.0001*
% Insulin		36.9	81.9	*<0.0001*
% On 1 injection of insulin		7.4	5.4	0.054
% On 2 or 3 injections of insulin		12.5	6.9	*<0.0001*
% On multiple injections of insulin		16.9	69.6	*<0.0001*
Mean BMI (kg/m^2^)		31.7 ± 5.8	31.2 ± 5.8	*0.009*
% Hypertension		55.6	49.9	*0.018*
Mean TG		192.5 ± 217.2	152.0 ± 144.4	*<0.0001*
Mean LDL		107.3 ± 37.4	77.9 ± 31.1	*<0.0001*
% LDL not on target in primary CV prevention		58.3	27.7	*<0.0001*
% LDL not on target in secondary CV prevention		78.7	44.8	*<0.0001*
% Smoking		17.2	16.6	0.709
% ≥2 CV risk factors		87.6	81.7	*<0.0001*
% DRP		27.2	40.2	*<0.0001*
% Microalbuminuria		28.1	34.4	*0.003*
% Carotid surgery		0.7 (6)	0.9 (8)	0.500
% Surgery lower limbs		3.7 (33)	6.9 (61)	*<0.0001*
% TIA/CVA		3.6 (32)	7.2 (64)	*<0.0001*
% Cardiac event		12.8 (113)	20.1 (178)	*<0.0001*
% CV event (peripheral; heart)		21.6 (191)	37.8 (335)	*<0.0001*
% New surgery lower limbs	2.6 (23)			
% New TIA/CVA	1.0 (9)			
% New cardiovascular event	4.1 (36)			

Data are mean ± SD, % or (number)

p values highlighted in italic are considered significant

*MF* metformin with or without insulin; *SU* sulfonylureum with or without insulin; *DPP-4* DPP-4-inhibitor; *GLP-1* GLP-1-receptor-agonist; *BMI* body mass index; *TG* triglycerides; *LDL* LDL-cholesterol; *CV* cardiovascular; *DRP* diabetic retinopathy; *TIA* transient ischemic attack; *CVA* cerebrovascular accident; *cardiac event* a myocardial infarction or an intervention such as a percutaneous transluminal coronary angioplasty or a coronary artery bypass graft

### Comparison of characteristics between the young- and old-onset T2DM cohorts at last contact

Compared to the old-onset T2DM cohort, patients with young-onset T2DM had a higher HbA1c [7.3 ± 1.3% (56 mmol/mol ± 14) vs. 6.9 ± 1.0% (51 mmol/mol ± 11), p < 0.0001] and smoked more often (16.6 vs. 7.0%, p = <0.0001). When adjusted for confounders (smoking, HbA1c, BMI, LDL-cholesterol, systolic and diastolic BP, age, diabetes duration and microalbuminuria), there was no difference in cardiovascular events between both groups (Table 5).

**Table 5:** Comparison of characteristics and complications between the young- and old-onset T2DM cohorts at last contact

	Age at diagnosis <45 years N = 886	Age at diagnosis 60–70 years N = 933	p value	Adjusted p value
% Men	55.9	53.2	0.246	
% Belgian nationality	91.3	97.2	*0.024*	
Mean age at last visit (years)	57.3 ± 12.6	75.7 ± 6.8	*<0.0001*	
Median age at diagnosis (years)	39.0 (8;45)	64.1 (60;70)	*<0.0001*	
Median follow-up (years)	7.6 (0;17)	4.6 (1;17)	*<0.0001*	
Mean diabetes duration (years)	20.5 ± 11.8	11.9 ± 6.6	*<0.0001*	
Mean HbA1c % (mmol/mol)	7.3 ± 1.3 (56 ± 14)	6.9 ± 1.0 (51 ± 11)	*<0.0001*	
Mean BMI (kg/m^2^)	31.2 ± 5.8	29.6 ± 5.5	*<0.0001*	
% Overweight	32.4	38.3	*0.011*	
% Obese	56.8	42.0	*<0.0001*	
% Hypertension	49.9	50.2	0.905	
Median TG	118.5 (25;2837)	118.0 (24;706)	0.829	
Mean LDL	77.5 ± 30.9	75.2 ± 29.4	0.163	
% Smoking	16.6	7.0	*<0.0001*	
% DRP	40.2	23.7	*<0.0001*	
% Microalbuminuria	34.4	34.0	0.915	
% Carotid surgery	0.9 (8)	4.3 (40)	*<0.0001*	0.843
% Surgery lower limbs	6.9 (61)	10.5 (98)	*0.006*	0.327
% TIA/CVA	7.2 (64)	16.1 (150)	*<0.0001*	0.739
% Cardiac event	20.1 (178)	31.8 (297)	*<0.0001*	0.386

Data are mean ± SD or %

p values highlighted in italic are considered significant

*BMI* body mass index; *TG* triglycerides; *LDL* LDL-cholesterol; *DRP* diabetic retinopathy; *TIA* transient ischemic attack; *CVA* cerebrovascular accident; *cardiac event* a myocardial infarction or an intervention such as a percutaneous transluminal coronary angioplasty or a coronary artery bypass graft

## Discussion

This study on a large Belgian cohort of adults diagnosed with T2DM <45 years provides insight in the general characteristics, complications and current care of this high-risk population. This study demonstrates that, despite a relatively young age at the last follow-up visit, young-onset T2DM patients have a high-risk phenotype and have a high life-time risk for cardiovascular complications.

In our young-onset T2DM cohort, 81.7% of patients had at least two cardiovascular risk factors at the last follow-up, a finding which is in agreement with the SEARCH study where 92% of T2DM patients had at least two cardiovascular risk factors [15]. Obesity is one of the main driving factors for the increase in prevalence of young-onset T2DM [1, 3]. Of our entire young-onset T2DM cohort, 32.4% of patients were overweight and 56.8% were obese. Compared to the first visit at our center, patients’ metabolic control improved significantly over time, but the frequency of cardiovascular risk factors remained high. This confirms the high-risk cardiovascular phenotype, as seen in other populations with young-onset T2DM [5, 8, 15–17]. The high prevalence of cardiovascular risk factors is alarming, since mortality in people with T2DM is mainly driven by cardiovascular disease and the incidence of macrovascular events increases with disease duration [1]. Early and aggressive risk factor management is therefore mandatory, but this is often very challenging to achieve as seen in our study [18, 19]. Cardiovascular risk factors remained often insufficiently controlled despite the frequent use of statins and antihypertensive medication in our population.

Data on long-term cardiovascular follow-up and mortality are limited in this population, in part because young-onset T2DM is a relatively new occurrence in European populations. A 9-year follow-up study of a very high-risk population of 69 First Nation Canadians adolescents with T2DM showed a mortality rate of 9% [20]. Our data confirm a high rate of cardiovascular complications in young-onset T2DM at a relatively young age, with 6.9% having received vascular surgery of the lower limbs, 7.2% having experienced a CVA and 20.1% having had a myocardial infarction or cardiac surgery by the time of the last follow-up visit. In our study, the rate of cardiac events remained significantly higher in men compared to women after adjustment for confounders. This is in contrast with findings of Hillier et al. [10] showing that the increased relative risk for myocardial infarction in young adults almost entirely occurred in women. This difference may be due to the fact that this study looked at complications emerging within 4 years after diagnosis in an American population, while our European study registered all cardiac events within a mean diabetes duration of 20.5 years which allowed sufficient time for those complications to occur. Studies from a large diabetes center in Sheffield in the UK demonstrated that diabetes duration was a significant predictor for microvascular and cardiovascular complications in young adults with T2DM, while age of diagnosis did not appear to be an important factor [9]. This is in line with the present study, since after adjustment for diabetes duration, age and cardiovascular risk factors, there was no difference in the rate of cardiovascular events between young-onset and old-onset diabetes patients. This would suggest that the more aggressive phenotype in young-onset T2DM is mostly due to the longer diabetes duration, with a comparable complication burden occurring at an earlier age [9]. However, this is in contrast with the data of Hillier et al. [1] showing that already at the time of diagnosis, adults with young-onset T2DM were more obese and had slightly worse glycemic control than the old-onset group. Furthermore, after controlling for length of follow-up (this equals diabetes duration since the study only included patients newly diagnosed with T2DM) young-onset T2DM patients had a 14-fold higher overall hazard of developing myocardial infarction compared to control subjects, while this was only a fourfold increased hazard in the old-onset T2DM group [10]. These data would therefore rather support the hypothesis that young-onset T2DM builds on a somewhat different pathogenesis (such as a more rapid decline in beta cell function and fructose in soft drinks increasing insulin resistance, lowering HDL and increasing triglycerides) resulting in an inherently more aggressive phenotype [2, 7, 9].

Achieving and maintaining good glycemic control seems to be more difficult in this young-onset T2DM population [9, 10, 18, 21]. In our cohort, glycemic control improved significantly over time, but more than half of patients still had an HbA1c above target at the last contact, despite 81.9% receiving insulin. This may be related to the fact that diagnosing diabetes mellitus at a younger age is associated with poor treatment adherence by patients and less aggressive treatment by health care professionals [9, 10, 18, 21]. However, there is few data to guide treatment of T2DM in youth. The ‘Treatment Options for type 2 Diabetes in Adolescents and Youth’ (TODAY) study was designed to compare the efficacy of three treatment regimens (metformin alone, metformin plus rosiglitazone and metformin plus intensive lifestyle modification) to achieve durable glycemic control in young-onset T2DM [19]. However, failure rates for all treatment arms were high [19].

In contrast to other series of T2DM in youth [3], there was a male predominance of T2DM in this cohort. Compared to women at their last visit, men had worse glycemic control despite a shorter diabetes duration. Although there was no significant difference in the frequency of insulin use, insulin might have been initiated earlier in women of childbearing age. Furthermore, men had more often at least three cardiovascular risk factors. In contrast, LDL-cholesterol was lower in men, probably due to the greater statin use compared to women. This might be related to the potential teratogenic effect of statins in women of childbearing age. These findings are in contrast with a recent study from two large diabetes centers in the UK showing equally prevalent risk factors between men and women [8].

An EM background is a known risk factor for the development of T2DM at a younger age. In particular, Japanese, Hispanic and Native American patients carry the highest risk of developing T2DM in childhood [5, 6]. In our cohort, 12.1% of patients had an EM background, which is in line with the frequency of EM in the background Belgian population [11]. In our cohort, the EM group had a similar metabolic control as the Caucasian population and were more often overweight but less often obese. This is in contrast with many studies showing that obesity disproportionally affects minorities [22]. This is probably due to the small absolute number of EM patients in our cohort.

Different studies report variable rates of microvascular complications in young-onset T2DM, probably due to differences in subjects’ age and disease duration [23]. A population-based cohort study in Sweden found similar rates of retinopathy between the T2DM and T1DM groups but the incidence of severe retinopathy was significantly higher in younger adults aged 15–34 years with T2DM compared with T1DM both at the time of diagnosis and after 10 years of follow-up [24]. In our cohort, 40.2% of patients had diabetic retinopathy at their last visit, with a high screening rate (91%). The prevalence of microalbuminuria at last visit in our young-onset population was 34.4%. This is comparable to data of Hillier et al. showing that about one in four adults developed incident microalbuminuria during follow-up, with similar findings in the young-onset group versus the usual-onset group. However, when adjusted for gender and length of follow-up, adults with young-onset T2DM had a 20% increased hazard of developing microalbuminuria compared to the usual-onset group [10].

The strengths of our study are the detailed analysis of a large cohort of young-onset T2DM patients and long-term follow-up data on cardiovascular complications. Limitations are the retrospective nature of the analysis, the lack of data on mortality and the fact that the first visit in our center was often many years after the diagnosis. Furthermore, this study is based on specialist care populations and the results might therefore not be extrapolated to patients followed in primary care. Another limitation is the use of different methods for data extraction concerning microalbuminuria and diabetic retinopathy in the young- versus old-onset T2DM groups.

## Conclusions

In conclusion, this study shows that young-onset T2DM is a prevalent condition with often insufficient metabolic control. Young-onset T2DM patients have a high rate of cardiovascular complications at a relatively young age. In our cohort, especially men were at a high risk for a cardiac event. Therefore, there is need for tailored treatment strategies to better manage this high-risk group. More research is also required to better understand how the pathophysiology of T2DM differs by the age of onset.

## Abbreviations

BMI: body mass index
BP: blood pressure
CABG: coronary artery bypass graft
CV: cardiovascular
CVA: cerebrovascular accident
DPP-4: DPP-4-inhibitor
DRP: diabetic retinopathy
EM: ethnic minority
GLP-1: GLP-1-receptor-agonist
LDL: LDL-cholesterol
MF: metformin with or without insulin
OAD: oral antidiabetic drugs
PTA: percutaneous transluminal arterioplasty
PTCA: percutaneous transluminal coronary angioplasty
SU: sulfonylureum with or without insulin
T1DM: type 1 diabetes
T2DM: type 2 diabetes
TG: triglycerides
TIA: transient ischemic attack
